# P-755. Comparison of Clinical and Safety Outcomes with Adjunctive Linezolid Versus Clindamycin in the Treatment of Necrotizing Soft Tissue Infections

**DOI:** 10.1093/ofid/ofaf695.966

**Published:** 2026-01-11

**Authors:** Andrew Matlob, Jing Zhao, Marco R Scipione, Alex Huang, Shannon Olson

**Affiliations:** DMC Harper University Hospital, Detroit, Michigan; Detroit Medical Center-Harper Hospital, Detroit, Michigan; Detroit Receiving Hospital, Detroit, MI; Detroit Medical Center, Detroit, Michigan; Sinai-Grace Hospital Detroit Medical Center, Detroit, Michigan

## Abstract

**Background:**

With decreasing susceptibility of *Streptococcus pyogenes* (GAS) to clindamycin, the Detroit Medical Center (DMC) has adopted linezolid as the toxin suppressive agent in the empiric treatment of necrotizing soft tissue infections (NSTIs). Given limited clinical data with linezolid, we evaluated clinical and safety outcomes between the use of linezolid vs clindamycin in the treatment of NSTIs.

**Methods:**

Retrospective cohort study of patients treated for NSTIs with adjunctive clindamycin from 8/2016 to 8/2018 or linezolid from 8/2022 to 8/2024 at DMC. All patients underwent surgery within 24 hours of admission and received susceptible therapy. Data collected included demographics, comorbidities, surgical data, infection site, antimicrobial therapy, cultures, clinical outcomes, and adverse events.

**Results:**

Of 72 eligible patients, 34 (47%) received clindamycin and 38 (53%) linezolid. Mean age 53±14 years, 71% male, 68% African American, 90% admitted from home, Charlson Comorbidity Index 3 [interquartile range (IQR) 0.5-5.5]. Patients in the linezolid group had a higher Laboratory Risk Indicator for Necrotizing Fasciitis (LRINEC) score [6 (IQR 4-8) vs 5 (IQR 4-6), p=0.05]. 19.4% had > 1 site of infection. Common sites: extremities (41.7%), genital/inguinal (36.1%), and gluteal/sacral (26.4%). Polymicrobial infection in 85.1% of cases; 32.9% had bacteremia. Organisms included anaerobes (77.6%), non-GAS (49.3%), gram-negative bacilli (37.3%), MSSA (11.9%), MRSA (10.4%), and GAS (3.0%). Time to surgery did not differ between groups [linezolid 5.3 hours (IQR 3.5-7.8) vs clindamycin 4.3 hours (IQR 1.3-8.8), p=0.3]. Duration of toxin suppressive therapy was longer with linezolid [5.4 days (IQR 3.5-8.0) vs 3.3 days (IQR 2.7-5.6), p=0.004]. Thirty-day mortality was 7.9% in the linezolid group vs 11.8% in the clindamycin group (p=0.4, OR=0.5, 95% CI 0.1-2.8). No significant difference in rates of thrombocytopenia (23.7% vs 14.7%, p=0.34) or *Clostridioides difficile* infection (0% vs 2.9%, p=0.29). Acute kidney injury (AKI) was significantly less with linezolid (5.3%) vs clindamycin (23.5%, p=0.03).

**Conclusion:**

Adjunctive linezolid may be a viable option for NSTIs and is associated with lower incidence of AKI. Further prospective randomized studies focusing on GAS are warranted.

**Disclosures:**

All Authors: No reported disclosures

